# TRBC1在成熟T细胞淋巴瘤中的表达特点及诊断价值

**DOI:** 10.3760/cma.j.issn.0253-2727.2022.07.008

**Published:** 2022-07

**Authors:** 宁涵 张, 肖 陈, 四书 赵, 纯 乔, 露 刘, 建勇 李, 雨洁 吴

**Affiliations:** 南京医科大学第一附属医院（江苏省人民医院）血液科，南京 210029 Department of Hematology, The First Affiliated Hospital of Nanjing Medical University, Jiangsu Provincial Hospital, Nanjing 210029, China

**Keywords:** 淋巴瘤，T细胞, TRBC1, 流式细胞术, Lymphoma, T-cell, TRBC1, Flow cytometry

## Abstract

**目的:**

分析TCRβ链恒定区结构域蛋白TRBC1在成熟T细胞淋巴瘤（TCL）中的表达特点，并与TCRVβ分析和TCR基因重排结果比较，探索TRBC1在TCL诊断中的价值。

**方法:**

通过多参数流式细胞术（FCM）检测南京医科大学第一附属医院血液科收治的30例TCL患者（TCL组）、40名正常对照和50例无T淋巴细胞增殖性疾病患者（非TCL组）TRBC1的表达特点，同时分析TCRVβ受体库检测、TCR基因重排与TRBC1限制性表达检测在TCL中的诊断价值。

**结果:**

正常对照组CD4^+^T和CD8^+^T细胞亚群TRBC1阳性表达率分别为（39.6±6.5）％和（39.3±4.4）％，非TCL组患者CD4^+^T和CD8^+^T细胞亚群TRBC1阳性表达率分别为（39.1±3.8）％和（36.0±8.4）％，TRBC1表达在两组中均呈双相表达模式。TCL组患者均为CD3^+^TCRγδ^−^，TRBC1阳性表达率>92.3％或<12.7％，所有病例均呈限制性表达模式（单克隆表达），与正常对照和非TCL组相比差异有统计学意义（*P*<0.001）。在T细胞克隆性检测效能方面，TRBC1灵敏性为100％，TCR基因重排阳性检出率为92.8％，TCRVβ流式试剂盒敏感性为94.1％，Kappa检验显示，三种检测方法一致性较高。

**结论:**

多参数FCM检测TRBC1表达水平能够快速高效地诊断TCL，具有较好的临床应用价值。

## Abstract (en)

**Objective:**

To investigate the expression characteristics of TRBC1 protein in mature T-cell lymphoma（TCL）, and compare with T-cell receptor（TCR）-Vβ repertoire analysis and TCR gene rearrangement results, to explore the value of TRBC1 in the diagnosis of TCL.

**Methods:**

The expression of TRBC1 was detected by multi-parameter flow cytometry in 30 cases of TCL, 40 cases of normal controls and 50 cases of patients without T lymphocyte proliferative diseases（non-TCL）admitted to the Department of Hematology, The First Affiliated Hospital of Nanjing Medical University. The diagnostic value of TCRVβ repertoire analysis, TCR gene rearrangement and TRBC1 restricted expression detection in TCL was evaluated.

**Results:**

The positive rates of CD4^+^T and CD8^+^T cell subsets TRBC1 in normal control group were（39.6±6.5）％ and（39.3±4.4）％. The positive rates of CD4^+^T and CD8^+^T cell subsets TRBC1 in non-TCL were（39.1±3.8）％ and（36.0±8.4）％. All 30 cases of TCL were CD3^+^TCRγδ^−^, and the positive rate of TRBC1 was >92.3％ or <12.7％. All cases showed restrictive expression pattern（monoclonal expression）, which was significantly different from those of the normal control and the non-TCL cases（*P*<0.001）. In terms of the diagnostic performance of T cell clonality, the sensitivity of TRBC1 was 100％, the positive detection rate of TCR gene rearrangement was 92.8％, and the sensitivity of TCRVβ detection was 94.1％. Kappa test showed high consistency among the three detective methods.

**Conclusion:**

Multi-parameter flow cytometry detection of TRBC1 expression level can quickly and efficiently diagnose mature T-cell lymphoma, which has good clinical application value.

成熟T细胞淋巴瘤（mature T cell lymphoma，TCL）是一类异质性血液系统恶性肿瘤，特点为T淋巴细胞的克隆性增殖[1]。常用的实验室诊断方法包括病理学、细胞形态学、分子生物学、流式细胞术免疫表型分析等。发现T细胞的单克隆表达是诊断TCL的重要依据，目前T细胞受体（TCR）基因重排和TCRs二代测序是评估T细胞克隆性的分子学方法[2]–[3]，但由于操作复杂、报告解读专业性高等因素，其临床推广受到一定限制。目前也有实验室采用流式细胞术（FCM）检测TCRβ链可变区（TCRVβ）受体库，共有24种亚家族，可以覆盖70％ T细胞克隆的亚家族。虽有报道显示该方法检测T细胞肿瘤灵敏度为89％，特异性为88％[4]–[5]，但由于成本要求较高，且覆盖面有限，在常规实验室推广应用有一定困难。近年来有研究发现一种针对人TCRβ链恒定区结构域（TRBC1）的单克隆抗体，可快速评估T细胞克隆的免疫表型，对TCL特异性较高[6]。本研究回顾性分析南京医科大学第一附属医院30例确诊TCL的免疫表型特征，并与TCR基因重排及TCRVβ受体库检测结果比较，探索TRBC1在TCL与非TCL中的表达差异及其在临床诊断中的价值。

## 病例与方法

1. 病例：纳入2021年1月−7月在南京医科大学第一附属医院（江苏省人民医院）血液科住院的30例TCL患者作为TCL组，纳入50例无T淋巴细胞增殖性疾病患者作为非TCL组。综合患者的临床症状、细胞形态学、免疫表型、组织病理学及影像学等检查结果，疾病诊断均符合2016年世界卫生组织造血和淋巴肿瘤分类修订版[7]–[8]。随机选取同时期40名体检健康者的外周血样本作为正常对照组。

2. FCM免疫分型：无菌操作下抽取2 ml骨髓或外周血，EDTA抗凝，在24 h内进行FCM检测。应用多参数FCM分析各组细胞表面不同抗原的表达情况，单克隆荧光抗体组合方案：CD3-PerCP-Cy5.5/CD4-PE/CD8-APC-Cy7/CD2-PE-Cy7/CD5-V450/CD7-APC/CD45-BV605/TCRγδ-APC-A750/TRBC1-FITC，所有抗体均购自美国Beckman公司。抗体与标本混匀后，室温下避光孵育20 min，用氯化铵溶血素裂解红细胞，PBS洗涤后上机检测。用美国Beckman Navios流式细胞仪收集100 000个细胞，以CD45/SSC、CD3/SSC等散点图设门，结合各个抗原的表达设门异常CD3^+^ T淋巴细胞，使用Kaluza Version 2.1（美国Beckman公司）分析目标细胞群体的免疫表型特点。

3. 毛细管电泳法BIOMED-2标准化TCR基因重排检测：用Ficoll-Hypaca分离外周血或骨髓单个核细胞。通过QIAamp试剂盒（德国Qiagen公司）提取和纯化DNA。采用超微量分光光度计（美国Thermo Scientific公司）检测DNA浓度与纯度。按照基因重排试剂盒（美国Invivoscribe公司产品）说明书进行操作。应用生物系统VeriTi热循环器（美国Applied Biosystems公司）对TCR Vβ、Dβ、Jβ、Vγ、Jγ片段进行PCR扩增，反应条件为：95 °C反应7 min，95 °C反应35 s，60 °C反应45 s，72 °C延伸90 s，共35个循环，最后72 °C延伸10 min。将PCR扩增产物与甲酰胺及GeneS-can-500 LIZ（美国Applied Biosystems公司）混合，经95 °C反应2 min热变性，4 °C反应5 min后采用3500 Dx基因扫描分析仪（美国Applied Biosystems公司）进行基因片段扫描分析。将已知TCR重排阳性参考品作为阳性对照，正常人单核细胞作为阴性对照[9]。

4. 统计学处理：采用SPSS 19.0及GraphPad Prism 6.0统计分析软件进行数据分析，根据数据分布类型，正态分布资料采用“均数±标准差”表示，偏态分布资料采用“中位数（范围）”表示，正态分布资料比较采用*t*检验或单因素方差分析，偏态分布资料比较采用非参数检验。分类变量资料采用“率”或“构成比”表示，组间比较采用Kappa检验或*χ*^2^检验，*P*<0.05为差异有统计学意义。

## 结果

1.临床特征：30例TCL患者中，男17例（56.7％），女13例（43.3％），中位年龄52（12～77）岁。其中外周T细胞淋巴瘤非特指型（PTCL-NOS）17例，T细胞大颗粒淋巴细胞白血病（T-LGLL）5例，T细胞幼淋巴细胞白血病（T-PLL）5例，结外鼻型NK/T细胞淋巴瘤（ENKTCL）2例，ALK阴性间变大细胞T细胞淋巴瘤（ALK-ALCL）1例，骨髓浸润24例。50例非TCL患者中，男28例（56.0％），女22例（44.0％），中位年龄58（20～73）岁，其中巨幼细胞贫血1例，再生障碍性贫血（AA）2例，B淋巴细胞增殖性疾病（B-LPD）21例，多发性骨髓瘤（MM）7例，骨髓增生异常综合征（MDS）3例，急性髓系白血病（AML）16例。正常对照组中，男21例（52.5％），女19例（47.5％），中位年龄44（30～56）岁，既往无血液病病史。TCL组的WBC、HGB、PLT、淋巴细胞绝对计数（ALC）等临床特征与非TCL组相比差异均无统计学意义（*P*值均≥0.05），TCL组的WBC、HGB、PLT、ALC与正常对照组相比差异均有统计学意义（*P*值均<0.05）（表1）。

**表1:** 成熟T细胞淋巴瘤（TCL）组与非 TCL组及正常对照组基线临床特征比较

临床特征	TCL组（30例）	非TCL组（50例）	正常对照组（40例）	*P*_1_值	*P*_2_值
性别（男/女，例）	17/13	28/22	21/19	0.95	0.73
年龄［*M*（范围）］	52（12～77）	58（20～73）	44（30～56）	0.40	0.01
WBC［×10^9^/L，*M*（范围）］	28.6（2.2～529.5）	24.7（3.6～417.9）	5.9（3.9～12.1）	0.09	0.01
HGB［g/L，*M*（范围）］	108.0（43.0～157.0）	95.0（34.0～140.0）	132.0（129.0～155.0）	0.07	0.01
PLT［×10^9^/L，*M*（范围）］	155（28～219）	190（19～344）	188（109～320）	0.05	0.01
ALC［×10^9^/L，*M*（范围）］	21.9（0.2～151.5）	18.6（1.0～390.3）	2.5（0.9～5.1）	0.65	0.01
标本类型（骨髓/外周血，例）	23/7	27/23	0/40	0.04	0.01
骨髓浸润（例）	24	17	0	0.01	0.01

注：ALC：淋巴细胞绝对计数；*P*_1_：TCL组与非TCL组比较；*P*_2_：TCL组与正常对照组比较

2. TRBC1在正常对照组和非TCL组中的表达情况：采用FCM评估非TCL组（50例）和正常对照组（40名）CD4^+^T和CD8^+^T细胞亚群中TRBC1的表达。两组研究样本的CD4^+^T或CD8^+^T细胞均显示出TRBC1双相表达模式，即TRBC1高表达和TRBC1低表达两种类型群体（图1、2），高表达组间与低表达组间细胞分布比例的差异均无统计学意义（*P*值均>0.05）。正常对照组CD4^+^T和CD8^+^T细胞亚群TRBC1^+^表达率分别为（39.6±6.5）％和（39.3±4.4）％（*P*>0.05），非TCL组CD4^+^T和CD8^+^T细胞亚群TRBC1^+^表达率分别为（39.1±3.8）％和（36.0±8.4）％（*P*>0.05）。两组间CD4^+^和CD8^+^T细胞亚群的TRBC1^+^表达率非常接近（*P*>0.05），CD4^+^T细胞TRBC1^+^表达略高，CD2、CD5、CD7表达均在正常范围。

**图1:**
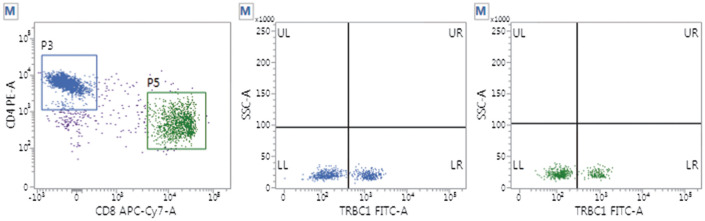
正常对照组TRBC1表达情况 蓝色：CD4^+^细胞；绿色：CD8^+^细胞

**图2:**
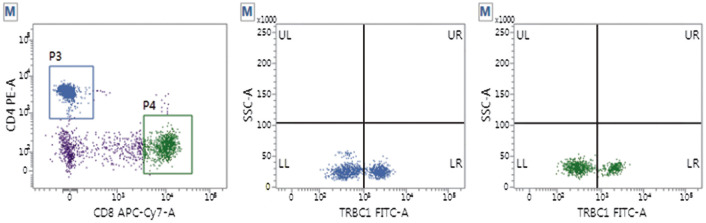
非成熟T细胞淋巴瘤组TRBC1表达情况 蓝色：CD4^+^细胞；绿色：CD8^+^细胞

3. TCL中TRBC1的表达情况：应用多参数FCM检测30例TCL患者的免疫表型，结果显示肿瘤T淋巴细胞均为CD3^+^TCRγδ^−^，TRBC1^+^表达率>92.3％或<12.7％，TCL组的TRBC1^+^表达率与正常对照组和非TCL组相比差异均有统计学意义（*P*值均<0.001）（图3）。

**图3:**
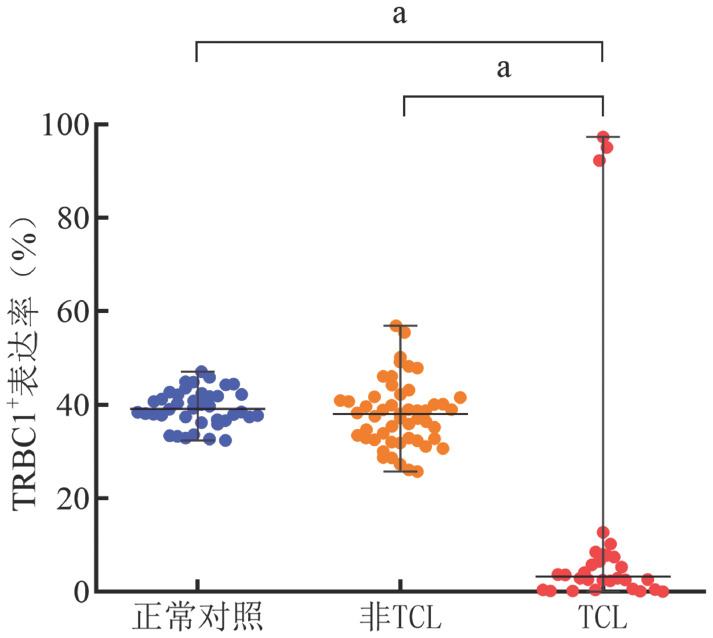
正常对照组、非TCL组和TCL组TRBC1^+^表达率 TCL：成熟T细胞淋巴瘤；^a^*P*<0.001

分析结果显示，克隆性T细胞呈TRBC1单相表达模式，高表达或低表达，灵敏性为100％。在30例TCL样本中，CD4^+^TCL 4例（13.3％），TRBC1表达率>95.7％或<12.7％（图4A、B）；CD4^+^CD8^+^TCL 4例（13.3％），TRBC1表达率<0.6％（图4C）；CD8^+^TCL 20例（66.7％），TRBC1表达率>92.3％或<8.5％（图4D、E）；CD4^−^CD8^−^TCL 2例（6.7％），TRBC1表达率<0.2％（图4F）。

**图4:**
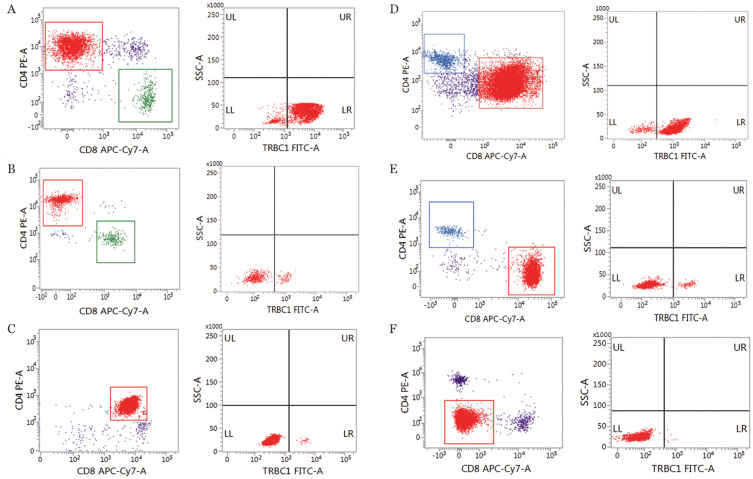
成熟T细胞淋巴瘤（TCL）中TRBC1表达情况 A、B：CD4^+^TCL中TRBC1表达情况；C：CD4^+^CD8^+^TCL中TRBC1表达情况；D、E：CD8^+^TCL中TRBC1表达情况；F：CD4^−^CD8^−^TCL中TRBC1表达情况。红色：肿瘤T细胞；蓝色：CD4^+^细胞；绿色：CD8^+^细胞

4. TCR基因重排分析和TCRVβ亚家族的表达特点：28例TCL中（其中2例未做TCR基因重排检测），TCRβ和TCRγ的阳性检出率分别为85.7％和78.6％，两者联合阳性检出率为92.8％，16例非TCL TCR基因重排均为阴性。17例TCL患者应用TCRVβ流式试剂盒进行检测，24种TCRVβ亚家族中呈限制性表达或阴性表达者16例，其中克隆性T淋巴细胞发生率最高的亚家族成员是TCRVβ13.2和TCRVβ5.2，TCRVβ亚家族呈均态表达者1例，敏感性为94.1％（图5）。

**图5:**
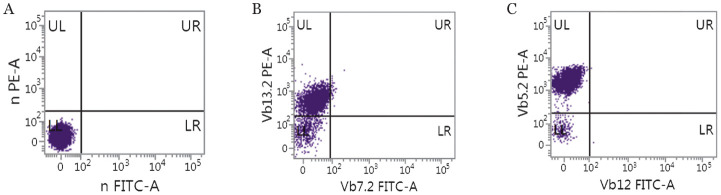
TCL组TCRVβ亚家族的表达模式 A：TCRVβ在对照管中呈正常表达模式；B：TCL组中TCRVβ13.2阳性率为85.1％，显著高于对照管（*P*<0.01）；C：TCL组中TCRVβ5.2阳性率为98.4％，显著高于对照管（*P*<0.01）

5. 比较三种检测方法对TCL的诊断价值：使用Kappa检验判断TRBC1检测、TCRVβ检测和TCR基因重排检测的结果是否一致。结果显示，Kappa值分别为0.86和0.95（*P*<0.01），说明三种方法的诊断结果存在一致性（表2）。在检测克隆性T细胞方面，三种方法具有相似的诊断效能。

**表2:** TRBC1检测、TCRVβ检测和TCR基因重排检测的一致性

TRBC1表达	TCRVβ检测		TCR基因重排	
	阳性	阴性	阳性	阴性
单克隆	16	1	27	1
多克隆	0	4	0	16

Kappa值	0.86		0.95	
*P*值	0.01		0.01	

## 讨论

TCL的诊断主要依赖细胞形态学、免疫表型和分子特征，如通过PCR检测TCR基因重排和FCM检测TCRVβ受体库等。由于上述方法价格昂贵且检测费时费力，很难推广，且需特殊试剂盒及仪器，难以在常规实验室开展，因此临床迫切需要一种快速准确的检测方法以优化当前诊断模式。TCR是成熟T细胞表面可识别并结合抗原的分子结构，TCRβ链恒定区域包括TRBC1和TRBC2两种基因[10]。正常细胞同时表达TRBC1^+^T细胞和TRBC2^+^ T细胞亚群，而TCL由于T细胞亚群克隆性增殖，仅表现为TRBC1^+^ T细胞或TRBC2^+^ T细胞单一亚型。由于目前尚无商品化TRBC2抗体，实际临床工作中通过检测TRBC1表达特点可判断是否存在表达异常，如检测TRBC1蛋白出现单型性或限制性表达，可作为诊断TCL的重要依据[11]。

在本研究中，我们采用包括TRBC1和T细胞相关9色抗体组合方案进行检测，发现TRBC1在肿瘤T细胞和正常T细胞中的表达存在显著差异，在非TCL组和正常对照组中CD4^+^T和CD8^+^T细胞亚群均显示出TRBC1双相表达模式，而在30例TCL中克隆性T细胞呈TRBC1单相表达模式。本研究表明，通过FCM检测肿瘤T细胞免疫表型是一种准确、可靠、简单的方法，可用于评估多种成熟T细胞肿瘤的克隆性。不仅是外周血和骨髓标本，还可通过检测淋巴结标本中的TRBC1免疫表型区分肿瘤T细胞和非肿瘤T细胞。TRBC1仅需一个抗体就能够覆盖所有CD3^+^TCL克隆性筛查，联合多个T系相关抗原进行设门和分析不仅更加灵活简便且节约标本和成本，更加适合临床推广。

临床工作中，TCR基因重排检测在T细胞克隆性检测中应用较为广泛。本研究采用TCR基因重排检测TCL克隆性的敏感性和特异性分别为89％和100％。基因扫描技术是TCR基因重排的一种分子学方法，相对简单灵敏，可检测0.5％～1％的单克隆T细胞，但该方法价格昂贵且假阳性率高[12]。另外，利用TCRβ或TCRγ重排阳性无法判断是αβ型还是γδ型TCL[9]。目前FCM检测TCRVβ受体库虽然可以能够检测24种TCRVβ亚家族，但也仅覆盖了70％左右的TCR受体亚家族。TRBC1检测TCR恒定区，因此稳定性更好，可以作为诊断T细胞克隆性的有效证据。需要注意的是，无论是TRBC1还是TCRVβ受体库检测方法，均限于检测TCRαβ亚型，不适用于TCRγδ^+^T细胞或CD3^−^细胞（如正常胸腺细胞、CD3阴性肿瘤和非常罕见的CD3阴性小反应亚群）的克隆性评估。在本研究中，1例外周T细胞淋巴瘤患者在有效治疗后TRBC1阳性率接近正常范围，提示TRBC1可用于αβTCL的治疗监测。有文献报道TRBC1抗体对成熟T细胞肿瘤的敏感性和特异性分别为97％和91％[13]。但在我们的研究中，TRBC1检测克隆性αβTCR亚群的敏感性为100％，我们认为是由于本研究采用9色抗体组合方案进行检测，因此更为敏感。本研究建议，通过TRBC1表达模式评估克隆性应采用定量和定性相结合的分析策略，需要更多更全面的含有TRBC1的T细胞抗体组合方案识别异常T细胞。值得一提的是，由于近期T细胞意义不明的克隆受到越来越多的关注[14]，需要更多色组合以利用不同免疫表型的表达特点综合分析，采用设门和反设门结合策略以发现可能隐藏在正常T细胞中的微小T细胞克隆。
